# Office of the Committee on Legislation and the Promotion of Homœopathy

**Published:** 1894-11

**Authors:** E. Francis Brady

**Affiliations:** Secretary of the Committee; Kansas City, Mo.


### OFFICE OF THE COMMITTEE ON LEGISLATION AND THE PROMOTION OF HOMŒOPATHY.

(Created and appointed by the Missouri Institute of Homœopathy, Session 1894.)

COMMITTEE:

T. H. HUDSON, M. D., Member State Board of Health, Kansas City, Mo., EX-OFFICIO CHAIRMAN.

W. B. MORGAN, M. D., President Missouri Institue Homœopathy, St. Louis, Mo.

H. J. RAVOLD, M. D., Gen'l Sec'y Missouri Institute Homœopathy, St. Joseph, Mo.

WM. C. RICHARDSON, M. D., St. Louis, Mo.

EDWARD F. BRADY, M. D., Secretary, Kansas City, Mo.

KANSAS CITY, MO., October 22d, 1894.

Editor of THE HOMŒOPATHIC PHYSICIAN:

The committee whose names appear at the head of this circular were created and appointed by the Missouri Institute of Homœopathy, at its recent annual session, held in St. Louis. In his annual address President Cutler referred to the fact that the Homœopathic School had no representation in any of the various State institutions, and called attention to the necessity for the Missouri Institute of Homœopathy, the recognized official body of our school, to take some action looking to the securing of legislation favorable to our interests, and the influencing of the executive head of the State, by petition and otherwise, to grant us such consideration as we may be able to show we are entitled to. A united effort with each homœopathic physician in the State exercising his personal influence, will undoubtedly secure for us an official recognition proportioned to our number and importance.

In order to accomplish the object aimed at, the committee have had printed the enclosed blank form of petition, to which you are requested to procure the signatures of as many citizens as possible, regardless of whether they are patrons of Homœopathy or not. The other blank is to be filled out with the data called for; our object in this latter blank is to disclose the fact of the number of persons in our population who patronize our system. This information is very important and will be a valuable aid to the committee in pushing their work before the governor or committee of the legislature. We think we will be able to get control of one of the Insane Asylums already under State management or else secure an additional one for Homœopathy; we also expect to have created a chair of Homœopathy in the State University at Columbia. While the conduct of this work has been delegated to the five physicians named, it is nevertheless the duty and responsibility of every homœopathic physician in this State to become interested in this movement for the advancement of our cause. If we sit down and fold our arms we must expect that our opponents will occupy every coign of vantage. But if we are alert, active, and persistent in urging our claims we will be certain to secure recognition.

The committee will be pleased to receive suggestions from all. Kindly acknowledge receipt of this circular letter and enclosures.

Fraternally yours,

E. Francis Brady,

*Secretary of the Committee.*

